# *In vitro* antioxidant activities of *Carissa edulis* ((Forssk) Vahl) and *Pappea capensis* (Eckyl. & Zeyh) extracts

**DOI:** 10.1016/j.heliyon.2023.e12965

**Published:** 2023-01-19

**Authors:** Carolyn Wanjira Muruthi, Mathew Piero Ngugi, Steven Maina Runo, Peter Githaiga Mwitari

**Affiliations:** a Department of Biochemistry, Microbiology and Biotechnology-Kenyatta University, Kenya; b Centre for Traditional Medicine and Drug Research-Kenya Medical Research Institute (KEMRI), Kenya

## Abstract

Herbal medications are gaining popularity due to their long history of use in traditional medicine. They serve as a reservoir for a diverse array of phytocompounds linked to amelioration of oxidative stress. Oxidative stress is a disturbance in the balance between generation and elimination of reactive species in human body. Moreover, reactive species are implicated in the onset and progression of chronic disorders. The current therapeutic approaches despite showing efficacy are characterized by several limitations such as adverse effects and prohibitive costs. This drives the need to explore alternatives that can inhibit, ameliorate or reverse conditions caused by oxidative stress. Several studies have evaluated antioxidant effects of diverse plant extracts. *C. edulis* and *P. capensis* are used as traditional therapy among the African communities to manage oxidative stress-related ailments. However, there is limited research on the antioxidant effects of these medicinal plants. The current study, therefore, sought to evaluate the antioxidant and phytochemical profile, of *C. edulis* and *P. capensis* extracts. Samples were collected from Embu County, Kenya. *In vitro* antioxidant properties of the extracts were evaluated through ferric reduction, Iron chelating, hydroxyl radical, and DPPH radical scavenging activities. Activities of catalase, superoxide dismutase and glutathione reductases of the extracts were further determined. Phytochemical profiles were determined using Liquid Chromatography Mass Spectrophotometer (LC-MS) and Gas Chromatography Mass Spectrophotometer (GC-MS) analyses. The extracts displayed concentration dependent antioxidant activities. Phytochemical analyses revealed presence compounds which are associated with antioxidant activities including flavonoids, phenolics, tocopherols and terpenoids. The findings provide a scientific validation for the folklore use of *C. edulis* and *P. capensis* in management of oxidative stress. Nevertheless, there is a need for further purification and characterization of phytochemicals associated with antioxidant activities.

## 1 Introduction

Herbal medications are gaining popularity due to their long history of use in traditional medicine [1]. Medicinal plants serve as a reservoir for a diverse array of phytocompounds linked to diverse pharmacological effects. Their bioactive components and metabolites, including phenolics, terpenoid and flavonoids, as well as their modified forms, found in the leaves, roots, barks and stems have shown substantial antioxidant potential [2]. Although the toxicity effects of a vast variety of medicinal plants has not been thoroughly explored, it is generally accepted that products from herb plants are safer compared to their synthetic counterparts [1].

Current studies link oxidative stress to onset and progression of chronic disorders including rheumatoid, cancer and diabetes [3,4]. Oxidative stress reflects a disturbance in the balance between generation and elimination of reactive species in human body [3]. ROS are the most significant free radicals in human body. They are unstable, reactive and partially reduced products of oxygen including superoxide anion and hydroxyl radical. Other reactive molecules in human body include reactive sulfide species and reactive nitrogen species [5]. Sources of free radicals can either be endogenous or exogenous [6]. Processes including mitochondrial respiratory chain, inflammation and cell-medicated immune activation are responsible for endogenous free radicals production. Exogenous reactive species production occurs due to exposure to radiation, heavy metals, environmental pollutants, cigarette smoke, pesticides and automobile exhaust fumes, among others [6].

As normal products of cellular metabolism, ROS are involved in cellular signaling and immune defence mechanisms. However, under a sustained environmental stress and failure of redox homeostasis, high levels of free radicals are generated and lead to oxidative alterations of proteins, lipids and nucleic acids. Cells deploy antioxidant ROS-scavenging systems mainly based on enzymatic components, including catalase, glutathione, superoxide dismutase (SOD). Further, small molecules viz. glutathione alpha-tocopherol, phenols and ascorbate acid, are part of the antioxidative defense systems [5]. However, intrinsic antioxidant defense systems may be overwhelmed by environmental and pathological factors [7]. Therefore, it is important to enhance body's natural antioxidant system.

Synthetic antioxidants including tertbutylhydroquinone (THQ) and butylated hydroxytoluene (BHT)) are used to remediate oxidative stressors. However, intake of synthetic antioxidants is challenged by their toxicity and potential health risks [7]. On the contrarily, plant based antioxidants continue to receive prominence since they are considered less expensive and abundant in several plant sources [8]. Antioxidant effects of plants extracts have been extensively investigated, with *Clerodendrum cyrtophyllum* [9], *Acacia catechu*, *Cinnamomum cassia* as well as *Citrus limon* extracts [10], *Calamintha officinalis,* [11] *Momordica charantia* and *Syzygium cumini* [12] exhibiting antioxidant activities.

*Carissa edulis* belongs to the Apocynaceae whereas *Pappea capensis*, the Sapindaceae family [13,14]. Diverse species in the Apocynaceae and Sapindaceae families have antioxidant activities. Among the Apocynaceae, Yadang et al. [15] demonstrated FRAP activities of aqueous, methanol and hydroethanol extracts of *C. edulis*. These effects were associated with phytocompounds including quercetin in the extracts. Similarly, reducing power activities, hydrogen peroxide and DPPH radical scavenging activities of *Carrisa carandas* have been documented [16]. Similarly, Ghagane et al. [17] documented that *Allophylus cobbe*, which belongs to the Sapindaceae family, has significant antioxidant activities that are attributed to presence of phenolics.

As medicinal plants, one of the hypothesized mechanistic approaches is through their antioxidant effect [18]. However, little information is available on the antioxidant effects of *C. edulis* and *P. capensis*. This study, therefore, focused on evaluating *in vitro* antioxidant activities of *C. edulis* and *P. capensis* extracts.

## 2 Materials and methodology

### 2.1 Collection and preparation of plant materials

The plant samples were collected from Embu County, Kenya with the assistance of a local herbalist. The plants were identified and authenticated by an acknowledged taxonomist, assigned voucher specimen's number and deposited at the Plant Science Departmental Herbarium, Kenyatta University. *C. edulis* was assigned voucher specimen number CM1 whereas *P. capensis* was assigned CM2. For the *in vitro* antioxidant activity assays, the plant material were sorted, cleaned and air dried in a shade for up to 20 days at room temperature. The dried samples were ground, powdered and stored awaiting extraction.

### 2.2 Extraction

For *in vitro* antioxidant activities aqueous and organic extraction was done. For aqueous extraction, a mass of 300 g of each of the pulverized plant samples was each soaked separately in 1 L of double distilled water. Extraction was carried out for 2hr in a water bath at 60 °C. The samples were set to cool, decanted, re-soaked and extracted for a further 2 h s in a water bath at 60 °C. The extracts were filtered and freeze-dried. The extracts were stored at 4 °C until use.

For organic extraction, three hundred grams of each powdered plant materials was soaked separately in 1 L of different solvents of EtOAc and DCM in a 2 L conical flask. The flasks were shaken regularly and left to stand for 48 h s. This was followed by filtration using Whatman filter paper No.1 and concentrated under a vacuum rotary evaporator at 400C. Each extract was stored until use at 4 °C.

For antioxidant enzyme activities, a mass 0.5 g of each plant material was separately homogenized with 5.0 ml of 100 mM potassium buffer (pH 7.0). This was performed under ice cold conditions. The mixture was then subjected to centrifugation for 20 min at 15,000 and supernatant was used for protein content and enzymes assays, including catalase, glutathione reductases and SOD.

### 2.3 Determination of antioxidant activities

#### 2.3.1 Ferric reducing antioxidant power

A modified procedure to evaluate FRAP activity of *C. edulis* and *P. capensis* was adapted from El Jemli et al. [19]. One ml of the sample was added to a mixture consisting of equal volume of phosphate buffer and potassium ferricyanide (2.5 ml) before incubation at 50 °C for 20 min. Thereafter, 2.5 ml (0.6 M) trichloroacetic acid (TCA) was added centrifuged (10min) at 3000 rpm. Finally, 2.5 ml of dH_2_O and 0.5 ml of ferric chloride were added to the supernatant and absorbance was read at 700 nm. Vitamin C was the reference compound whereas the control comprised of all reagents without extracts or standard. The experiment was undertaken in triplicate.

#### 2.3.2 DPPH free radical scavenging activity

DPPH scavenging activity was estimated based on the procedure described by Sahin et al. [20] with slight adjustments. In methanol, a solution (0.1 mM) of DPPH was prepared. An aliquot of 0.5 ml was added to 1 ml of sample at varying concentration (0.05, 0.1, 0.5, 1.0, 2.0, 5.0 mg/ml). A mixture of DPPH and methanol was used as control whereas ascorbic acid was the reference standard. A decrease in absorbance was read at 517 nm after incubation in the dark for 20 min. The experiments were run three times. Percentage scavenging activity was computed as follows (Sahin et al., 2004);

$$\%\;RSA=\frac{\left(Abs\;control-Abs\;sample\right)}{Abs\;control}\times 100$$

Where,

% RSA = Radical Scavenging Activity.

Abs control = absorbance of DPPH radical + methanol;

Abs sample = absorbance of DPPH radical + plant extract/DPPH radical + ascorbic acid.

The IC_50_ values of the samples were also determined.

#### 2.3.3 Iron (Fe^2+^) chelating activity

A slight modified protocol by Adjimani and Asare [21] was adopted in determining chelating activity on ferrous ions by *C. edulis* and *P. capensis.* One ml of the test compound was added to 2.5 ml of ferrous sulphate solution (0.125 mM) followed by an equal volume of ferrozine (0.3125 mM) before incubation for 10 min. The tests were done in triplicate whereas absorbance was read at 562 nm. EDTA was considered as the standard. A mixture of reagents without the sample or reference standard was used as control. Percentage chelating effect was computed using the equation previously described by Adjimani and Asare [21];

$$Chelating\;effect\;\left(\%\right)=\frac{Ac-As}{Ac}\times 100$$

Where,

Ac = Absorbance of control.

As = Absorbance of sample.

#### 2.3.4 Hydroxyl radical scavenging activity

A modified method by Rahman et al. [4] was used to evaluate the ability of *C. edulis* and *P. capensis* to scavenge hydroxyl radical. A final reaction mixture (1.0 ml) contained 200 μl of 15 mM of d-ribose in 200 μl of 100 KH_2_PO_4_–KOH potassium phosphate buffer (pH 7.4), 100 μl of extract or reference compound (gallic acid), 100 μl of EDTA and 200 μl of 500 mM FeCl_3_ ferric chloride, 100 μl of 1 mM ascorbic acid and 100 μl of H_2_O_2_. The mixture was incubated for 1 h, 1 ml each of thiobarbituric acid and trichloroacetic acid was thereafter added. This was followed by incubation for another 10 min at 100 °C. The mixture was cooled and absorbance was read at 700 nm. The experiment was performed three times. Scavenging activity was expressed in percentage using a formula by Rahman et al. (2015);

$$\%\;Hydroxyl\;radical\;scavenging\;activity=\frac{Abs\;Control\;–Abs\;Sample}{Abs\;Control}\times 100$$

Where;

Abs Control = Control absorbance.

Abs Sample = extract or standard absorbance.

### 2.4 Determination of antioxidant enzyme activity

#### 2.4.1 Protein estimation

##### 2.4.1.1 Reagents

Reagent A- 500 ml of alkaline solution (2.86 g NAOH, 14.31 g Na_2_CO_3_).

Reagent B-100 ml (1.42 g CuSO_4_. 5(H_2_O), 2.85 g C_4_H_4_Na_2_O_6_. 2(H_2_O).

Reagent C-50ml of reagent A and 1.0 ml of reagent B.

Reagent D- FC reagent protein solution.

Protein solution (stock standard) - 50 ml (50 mg of bovine serum (BSA) dissolved in dH_2_0 to a final volume of 50 ml.

##### 2.4.1.2 Procedure

Total protein estimation was done using conventional Lowry's method as previously described by Sarkar et al. [22]. Volumes of 0.1 ml of the extracts and standard were separately pipetted into a series of test tubes (10–100 mg/ml). Thereafter, to each test tube, 5.0 ml of reagent C and 0.5 ml of reagent D were added, followed by incubation for 30 min. Absorbance was recorded at 590 nm. The experiment was run in triplicate. A graph of absorbance versus concentration was plotted for standard solution. Concentrations of proteins in *C. edulis* and *P. capensis* extracts were estimated from the graph. Protein concentration extracts was expressed as mg/g FW (Sarkar et al., 2020).

#### 2.4.2 Catalase activity

A protocol by Sharma et al. [23] was employed to establish catalase activity of the plant extracts. To a reaction mixture consisting of 1.5 ml phosphate buffer and 300 μl plant extracts, 1.2 ml of hydrogen peroxide was added. The rate of H_2_O_2_ decomposition was indicated by a decrease in absorbance at 240 nm. Triplicate experiments were carried out and activity of the enzyme was computed by the following formula;

$$Enzyme\;activity\;\left(units/\min/\;gfw\right)=\frac{Change\;in\;abs/\min\;X\;total\;volume\;\left(ml\right)}{Ext.coefficient\;X\;volume\;of\;sample\;taken\;\left(ml\right)}$$

Where, extinction coefficient = 6.93 × 10-3 mM^−1^cm^−1^

Abs/min = absorbance per minute

$$Specific\;activity\;\left(mmol\;UA/mg\;protein\right)=\frac{Enzyme\;activity\left(\frac{unit}{minute}/gFW\right)}{Protein\;content\;\left(\frac{mg}{g}FW\right)}$$

#### 2.4.3 Superoxide dismutase (SOD) activity

Activity of SOD was conducted spectrophotometrically by a method adapted from Shama et al. (2007). Hydroxylamine hydrochloride (100 μl) was added to a mixture containing 1.3 ml sodium carbonate buffer, 100 μl TritonX-100 and 500 μl NBT. After 2min, 70 μl of the enzyme extract was then added. Absorbance was measured spectrophotometically at 540 nm. A control reaction was carried out using all reagents without the plant extracts. The experiment was carried out in triplicate. Inhibition of NBT reduction was computed using a formula described by Sharma et al. (2007);

$$Y=\frac{Change\;in\;abs/\min\left(blank\right)-change\;in\;abs/\min\left(sample\right)}{Change\;in\;absorbance/\min\left(blank\right)}$$

Where;

Abs/min = absorbance per minute.

Y = % inhibition produced by 70 μl of sample.

Hence, 50% inhibition is produced by 50 × 70/y = z μl of sample.

To calculate number of enzymes per unit as formulated as follows;

$$Enzyme\;activity\;\left(units/\min/\;gfw\right)=\frac{Change\;in\;abs/\min\times total\;volume\;\left(ml\right)}{Ext.coefficient\times volume\;of\;sample\;taken\;\left(ml\right)}$$

Where, extinction coefficient = 6.22mM^−1^cm^−1^

Abs/min = absorbance/minute

$$Specific\;activity\;\left(mmol\;UA/mg\;protein\right)=\frac{Enzyme\;activity\left(\frac{unit}{minute}/gFW\right)}{Protein\;content\;\left(\frac{mg}{g}FW\right)}$$

#### 2.4.4 Glutathione reductase assay

Glutathione reductase activity was done following a protocol previously described by Sharma et al. [23]. Reductase activity was determined by measuring oxidation of NADPH in a mixture containing 300 μl each of NADPH, enzyme extract, and oxidized glutathione (GSSG) and 1.8 ml phosphate buffer. All tests were done in triplicate. Decrease in absorbance per minute was recorded at 340 nm. The enzyme activity was computed according to the following equation;

$$Enzyme\;activity\;\left(units/\min/\;gfw\right)=\frac{Change\;in\;abs/\min\times total\;volume\;\left(ml\right)}{Ext.coefficient\times volume\;of\;sample\;taken\;\left(ml\right)}$$

Where, extinction coefficient = 6.22mM^−1^cm^−1^

Abs/min = absorbance per minute

$$Specific\;activity\;\left(mmol\;UA/mg\;protein\right)=\frac{Enzyme\;activity\left(\frac{unit}{minute}/gFW\right)}{Protein\;content\;\left(\frac{mg}{g}FW\right)}$$

### 2.5 Quantitative phytochemical profile

#### 2.5.1 Gas chromatography mass spectrophotometry

GC-MS analyses of the extracts were carried out using GC-MS instrument equipped with a BPX5 non-polar capillary column, 30 m; 0.25 mm ID; 0.25 μm film thickness. Carrier gas (pure Helium) was at a constant flow rate of at a flow rate of 1.25 ml/min in split mode. For spectral detection, ionization energy of 70 electron Volts was employed with mass fragment range from 50 to 600 *m*/*z*, and scan speed, 1666 μm/s. The column oven temperature was set at at 35 °C (5 min); for 10.5 min, the temperatures were increased (10 °C/min to 250 °C). This was followed by a further increase for 29.9 min at 285 °C.

#### 2.5.2 Liquid chromatography mass spectrophotometry

LC-MS analysis separation of *C. edulis* and *P. capensis* extracts was done using an ACQUITY UPLC BEH C18 column (Waters Corp., Wexford, Ireland). The mobile phase consisted of deionized water with 0.01% formic acid (A) and methanol (B). Gradient elution flow was set at 0.2 ml/min whereas the solvent gradient system initiated at as initiated at 5% B and a final 100% held for 20 min. The mass spectrometric conditions were follows; *m*/*z* range 40–2,000, desolvation temperature of 150 °C and 120 °C and the drying gas (nitrogen) ﬂow rate was set at 800 L/h.

The content of phytocompounds in the test samples were identified by comparing their retention time, peak height, peak area and mass spectral patterns with those of authentic compound found in the National Institute of Standards and Technology (NIST) library.

## 3 Statistical analysis

Absorbance measures on antioxidant activities of *C. edulis* and *P. capensis* were entered and organized in Microsoft Excel spreadsheet and exported to Graph Pad Prism 5 for analysis. Descriptive statistics were then undertaken and values expressed as mean ± SEM. One Way ANOVA was done to compare percentage radical scavenging activity among the extracts followed by a post hoc test (Tukey's) at 95% level of significance for pairwise comparison. Unpaired *t*-test was used to compare antioxidant activities of similar extracts. IC_50_ values were computed using linear regression analysis.

## 4 Results

### 4.1 Ferric reducing antioxidant power of *C. edulis* and *P. capensis* extracts

**Fig. 1:**
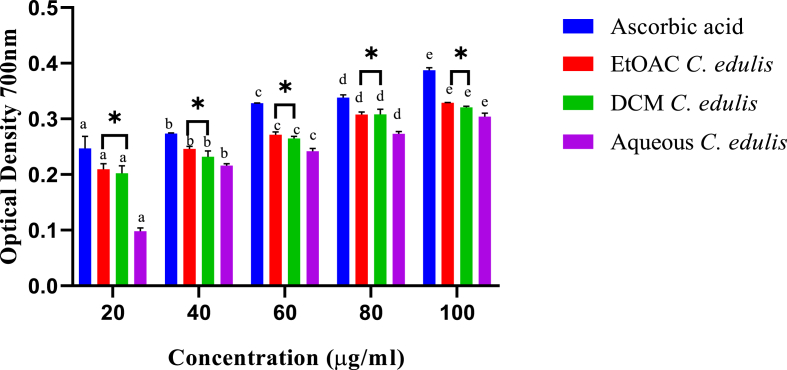
*In vitro* FRAP activity of leaf extracts of *C. edulis*. Bars with different lowercase are statistically significant (p < 0.05). Within the same concentration, bars with asterisk (*) are not statistically significantly (p > 0.05) by one way ANOVA followed by Tukey's post hoc test.

The ferric reducing capacity of *C. edulis* extracts showed an exponential reduction of ferric ions from the least to the highest extracts concentration (Fig. 5.1). Further, the ferric reducing capacities were significantly different across the five tested concentrations (p < 0.05; Fig. 1). The ability of ethylacetate and dichloromethane extracts to reduce ferric ions were comparable across all the tested concentrations (p > 0.05; Fig. 1). By contrast, the aqueous extract showed the least ferric reducing ability at all the tested concentration. The ability to reduce ferric ions by ascorbic acid (standard) was significantly higher compared to those of *C. edulis* extracts at all tested concentrations (p < 0.05; Fig. 1).

**Fig. 2:**
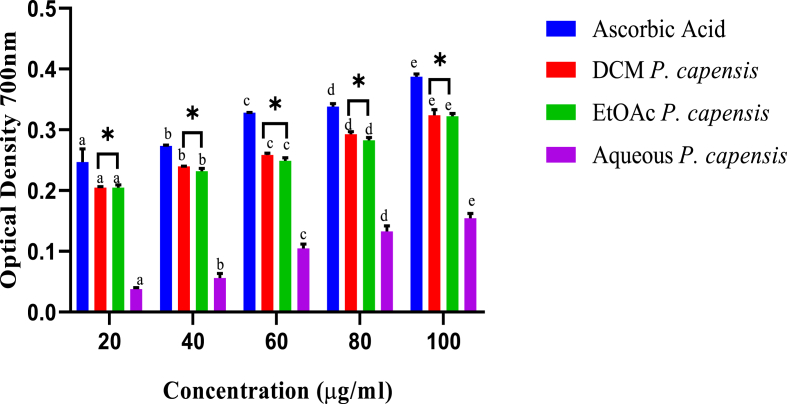
*In vitro* FRAP activity of stem bark extracts of *P. capensis.* Bars with different lowercase are statistically significant (p < 0.05). Within the same concentration, bars with asterisk (*) are not statistically significantly (p > 0.05) by one way ANOVA followed by Tukey's post hoc test.

Similarly, the ferric reducing powers of *P. capensis* extracts were concentration dependent (Fig. 2). Further, the ferric reducing activity of *P. capensis* extracts was statistically different among the five extracts concentrations (p < 0.05; Fig. 2). Moreover, at all the concentrations, the ferric reducing effects of ethylacetate extract were statistically comparable to those of the dichloromethane extract of *P. capensis* (p > 0.05). However, the ferric reducing effects of the standard, were significantly higher compared to those of *P. capensis* extracts across the tested concentrations (p < 0.05; Fig. 2).

### 4.2 Metal ions chelating activities of *C. edulis* and *P. capensis* extracts

**Fig. 3:**
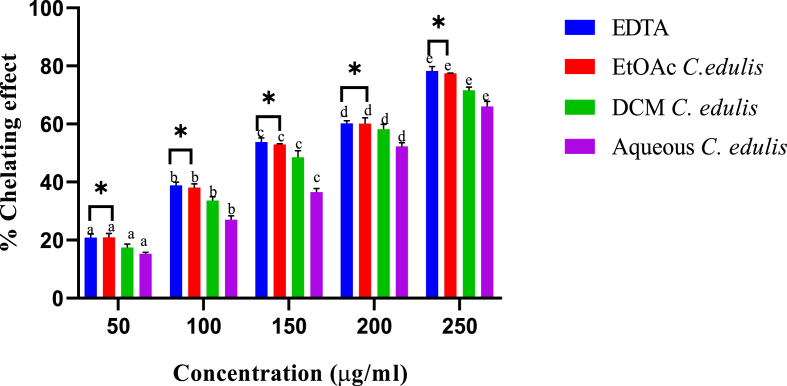
*In vitro* iron-chelating activity of leaf extracts of *C. edulis*. Bars with different lowercase are statistically significant (p < 0.05). Within the same concentration, bars with asterisk (*) are not statistically significantly (p > 0.05) by one way ANOVA followed by Tukey's post hoc test.

Extracts of *C. edulis* displayed potent chelating activity on ferrous ions. The ability by *C. edulis* extracts to chelate ferrous ions was concentration-dependent (Fig. 3). Among the five tested extracts concentrations the chelating activities were incomparable from each other (p < 0.05; Fig. 3).

As Fig. 3 shows, at all the tested concentrations, ferrous ions chelating effects of ethylacetate *C. edulis* extract were comparable to those of EDTA, a chelator (p > 0.05). Further analysis showed that the aqueous extract exhibited the lowest capacity to chelate ferrous ions (Fig. 3).

As shown in Table 1, the concentration of ethylacetate extract of *C. edulis* that inhibited ferrozine-Fe^2+^ complexes formation by 50% was 134.07 ± 0.47 μg/ml. On the other hand, the IC_50_ values of the effect of the dichloromethane and aqueous extracts were 151.77 ± 0.38 μg/ml and 183.30 ± 1.11 μg/ml, respectively. EDTA had an IC_50_ value of 132.23 ± 1.48 μg/ml (Table 1).

**Table 1:** The concentrations of *C. edulis* extracts needed to inhibit 50% of the radical

Radical Formed	Sample	IC_50_ (μg/ml)
Iron (II ferrozine complex)	*C. edulis* Aqueous	134.07 ± 0.47^a^
	*C. edulis* EtoAc	183.30 ± 1.11^c^
	*C. edulis* DCM	151.77 ± 0.38^b^
	EDTA	132.23 ± 1.48^a^

Results are expressed as mean ± SEM. Means with identical superscript are not statistically different at p > 0.05 by one way ANOVA followed by Tukey's post hoc test.

**Fig. 4:**
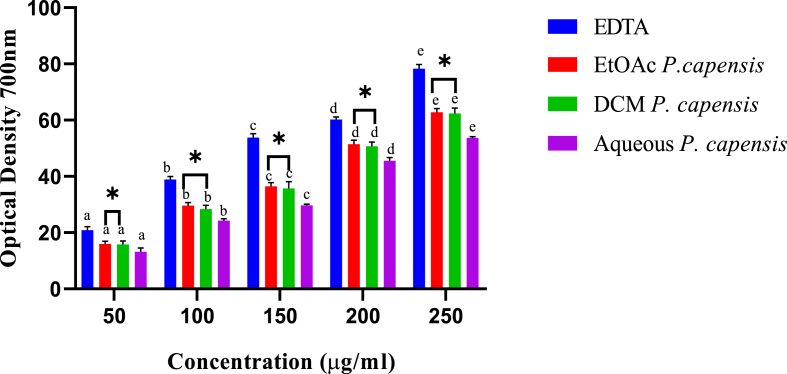
*In vitro* iron-chelating activity of stem bark extracts of *P. capensis.* Bars with different lowercase are statistically significant (p < 0.05). Within the same concentration, bars with asterisk (*) are not statistically significantly (p > 0.05) by one way ANOVA followed by Tukey's post hoc test.

Similarly, the capacity to chelate ferrous ions by aqueous, ethylacetate and dichloromethane extracts of *P. capensis* increased with the increase of extract concentration (Fig. 4).

In the five tested concentrations, the ethylacetate extract of *P. capensis* had statistically similar chelating effects with those of the dichloromethane extract. (p > 0.05) Moreover, EDTA exhibited significantly higher chelating activities compared to those of *P. capensis* extracts (p < 0.05; Fig. 4).

The IC_50_ values of *P. capensis* extracts were higher compared to that of EDTA (132.23 ± 1.48 μg/ml). The IC_50_ values of aqueous, ethylacetate and dichloromethane were 144.57 ± 3.60 μg/ml, 194.83 ± 3.49 μg/ml and 235.03 ± 0.33 μg/ml, respectively (Table 2).

**Table 2:** The concentrations of *P. capensis* extracts needed to inhibit 50% of the radical

Radical Formed	Sample	IC_50_ (μg/ml)
Iron (II ferrozine complex)	*P. capensis* Aqueous	144.57 ± 3.60^b^
	*P. capensis* EtoAc	194.83 ± 3.49^c^
	*P. capensis* DCM	235.03 ± 0.33^d^
	EDTA	132.23 ± 1.48^a^

Results are expressed as mean ± SEM for replicate measurements n = 3. Values with the identical superscript are not statistically different from each other at p > 0.05 by one way ANOVA followed by Tukey's post hoc test.

### 4.3 Hydroxyl scavenging radical activities of *C. edulis* and *P. capensis* extracts

**Fig. 5:**
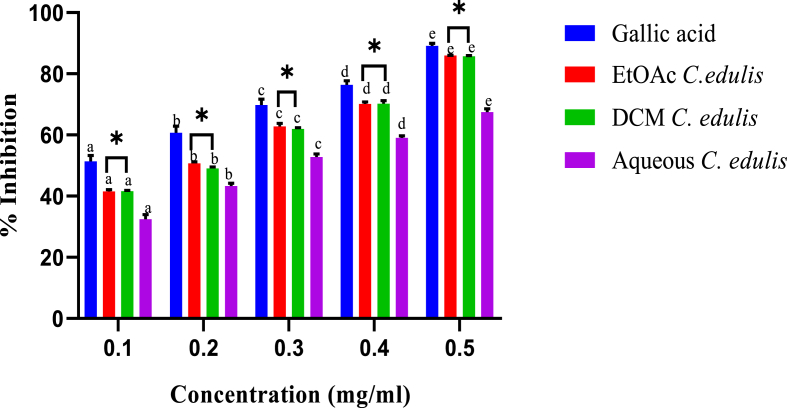
*In vitro* hydroxyl radical scavenging activity of leaf extracts *C. edulis*. Bars with different lowercase are statistically significant (p < 0.05). Within the same concentration, bars with asterisk (*) are not statistically significantly (p > 0.05) by one way ANOVA followed by Tukey's post hoc test.

The hydroxyl radical scavenging activity of *C. edulis* extracts is shown in Fig. 5. In each extract, maximum scavenging activity was observed at the highest extract concentration (Fig. 5). Hydroxyl scavenging potency of ethylacetate and dichloromethane leaf extracts of *C. edulis* were statistically comparable across in all the five extracts concentrations (p > 0.05) but statistically higher compared to those of the aqueous extract (p < 0.05; Fig. 5).

**Fig. 6:**
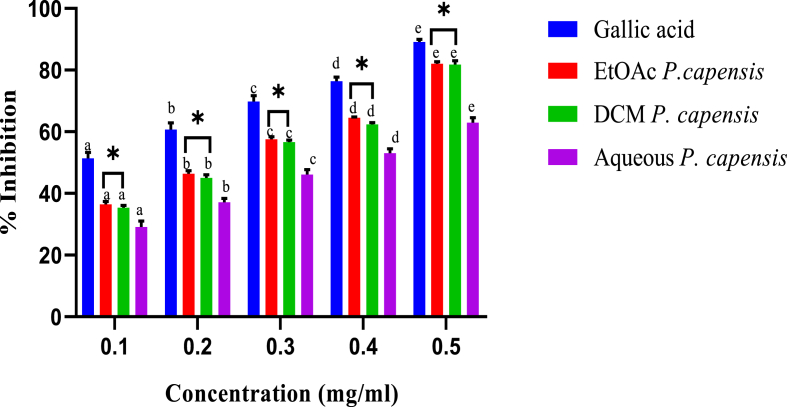
*In vitro* hydroxyl radical scavenging activity of stem bark extracts of *P. capensis.* Bars with different lowercase are statistically significant (p < 0.05). Within the same concentration, bars with asterisk (*) are not statistically significantly (p > 0.05) by one way ANOVA followed by Tukey's post hoc test.

From the analysis, the least amount of the ethylacetate extract of *C. edulis* required to elicit hydroxyl scavenging activity was 0.16 ± 0.02 mg/ml, whereas those of dichloromethane and aqueous extracts were 0.23 ± 0.05 mg/ml and 0.25 ± 0.04 mg/ml, respectively. Gallic acid showed the lowest IC_50_ value (0.13 ± 0.03 mg/ml) (Table 3). As shown in Fig. 6, the hydroxyl radical scavenging abilities of *P. capensis* extracts were concentration-dependent (Fig. 6). The highest extract concentration was more potent compared to the lower extract concentrations (Fig. 6).

**Table 3:** The concentrations of *C. edulis* extracts needed to inhibit 50% of the radical formed

Radical Formed	Sample	IC_50_ (μg/ml)
Hydroxyl radical	*C. edulis* Aqueous	250 ± 0.04^c^
	*C. edulis* EtoAc	160 ± 0.02^b^
	*C. edulis* DCM	230 ± 0.05^c^
	Gallic acid	130 ± 0.03^a^

Results are expressed as mean ± SEM. Means with the identical superscript are not statistically different at p > 0.05 by one way ANOVA followed by Tukey's post hoc test.

As shown in Fig. 6, in the five tested extract concentrations, the hydroxyl radical scavenging activities of ethylacetate and dichloromethane extracts were comparable (p > 0.05). However, the aqueous extracts of *P. capensis* showed the least activities, whereas gallic acid exhibited the highest hydroxyl radical scavenging activities at all concentrations (Fig. 6).

The current study also showed that the concentration required to inhibit formation of hydroxyl radical by 50% (IC_50_) by the ethylacetate extract of *P. capensis* was 0.20 ± 0.21 mg/ml, whereas those of dichloromethane and aqueous extracts were 0.21 ± 0.20 and 0.32 ± 0.19 mg/ml, respectively. Gallic acid showed the highest scavenging effect with an IC_50_ value of 0.13 ± 0.03 mg/ml (Table 4).

**Table 4:** The concentrations of *P. capensis* extracts needed to inhibit 50% of the radical

Radical Formed	Sample	IC_50_ (μg/ml)
Hydroxyl radical	*P. capensis* Aqueous	320 ± 0.19^c^
	*P. capensis* EtoAc	200 ± 0.21^b^
	*P. capensis* DCM	210 ± 0.20^b^
	Gallic acid	130 ± 0.03^a^

Results are expressed as mean ± SEM. Means with the identical superscript are not statistically significant at p > 0.05 by one way ANOVA followed by Tukey's post hoc test.

### 4.4 DPPH radical scavenging activities of *C. edulis* and *P. capensis* extracts

**Fig. 7:**
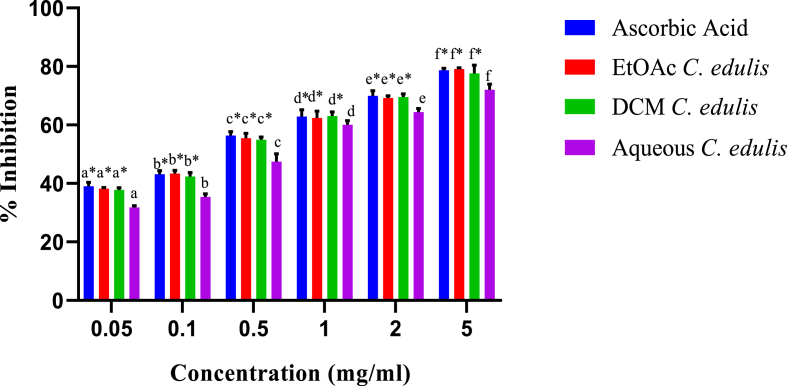
*In vitro* DPPH scavenging activity of leaf extract of *C. edulis.* Bars with different lowercase are statistically significant (p < 0.05). Within the same concentration, bars with asterisk (*) are not statistically significantly (p > 0.05) by one way ANOVA followed by Tukey's post hoc test.

The *C. edulis* extracts revealed a concentration-dependent DPPH free radical scavenging activity. Fig. 7 shows that the highest scavenging effect was observed at the highest extract concentration. Albeit the five extract concentrations revealed potent efficacy against DPPH radicals, the scavenging activity significantly differed from each other (p < 0.05). The DPPH radical scavenging activities of dichloromethane and ethylacetate of *C. edulis* extracts were statistically comparable to those ascorbic acid (p > 0.05; Fig. 7).

Furthermore, IC_50_ values the ethylacetate extract of *C. edulis* was 0.22 ± 0.06 mg/ml, whereas those of dichloromethane and aqueous extracts, 0.23 ± 0.20 and 0.43 ± 0.16 mg/ml, respectively Ascorbic acid had an IC_50_ value of 0.20 ± 0.01 mg/ml (Table 5).

**Table 5:** The concentrations of *C. edulis* extracts needed to inhibit 50% of the radical

Radical Formed	Sample	IC_50_ (μg/ml)
DPPH radical	*C. edulis* Aqueous	430 ± 0.16^b^
	*C. edulis* EtoAc	220 ± 0.06^a^
	*C. edulis* DCM	230 ± 0.20^a^
	Ascorbic acid	200 ± 0.01^a^

Results are expressed as mean ± SEM. Means with the identical superscript are not statistically significant at p > 0.05 by one way ANOVA followed by Tukey's post hoc test.

**Fig. 8:**
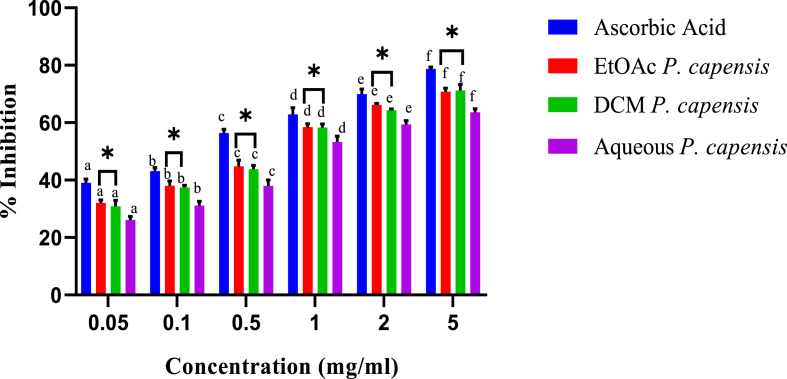
*In vitro* DPPH scavenging activity of stem bark extracts *P. capensis.* Bars with different lowercase are statistically significant (p < 0.05). Within the same concentration, bars with asterisk (*) are not statistically significantly (p > 0.05) by one way ANOVA followed by Tukey's post hoc test.

The current study also revealed that the DPPH radical scavenging activities of aqueous, ethylacetate and dichloromethane stem bark extracts of *P. capensis* were concentration-dependent (Fig. 8). In comparison, the DPPH radical scavenging effects of the ethylacetate extract of *P. capensis* were statistically similar to those of dichloromethane extract in all the five concentrations (p > 0.05; Fig. 8). However, ascorbic acid had a significantly higher activity compared to *P. capensis* extracts (p < 0.05; Fig. 8).

In terms of IC_50,_ the ethylacetate extract *P. capensis* extract had an IC_50_ value of 0.43 ± 0.06 mg/ml whereas the dichloromethane and aqueous extracts scavenged DPPH radical with half maximal inhibitory concentration values of 0.43 ± 0.02 mg/ml and 0.46 ± 0.03 mg/ml, respectively (Table 6).

**Table 6:** The concentrations of *P. capensis* extracts needed to inhibit 50% of the radical

Radical Formed	Sample	IC_50_ (μg/ml)
DPPH radical	*P. capensis* Aqueous	460 ± 0.16^c^
	*P. capensis* EtoAc	430 ± 0.06^b^
	*P. capensis* DCM	430 ± 0.02^b^
	Ascorbic acid	200 ± 0.01^a^

Results are expressed as mean ± SEM. Means with the identical superscript are not statistically significant at p > 0.05 by one way ANOVA followed by Tukey's post hoc test.

#### 4.4.1 Total protein content antioxidant enzyme activities of *P. capensis* and *C. edulis* extracts

In the present study, *C. edulis* and *P. capensis* extracts exhibited varying protein contents as shown in Table 7. The total protein content in the leaf extract of *C. edulis* extract was statistically higher compared to that of the *P. capensis* extract (p < 0.05). Additionally, the catalase activity of *C. edulis* extract was significantly higher compared to that of *P. capensis* extract (p < 0.05), whereby the leaf extract of *C. edulis* had catalase activity of 26.66 unit/m proteins, whereas that of the stem bark extract of *P. capensis* had catalase activity of 49.36 unit/mg proteins (Table 7).

**Table 7:** Total protein estimation and enzymatic activities of *C. edulis* and *P. capensis* extracts.

Plant	Total Protein Estimation (mg/ml)	Catalase Activity (Unit/mg Protein)	Superoxide Dismutase (SOD) Activity (Unit/mg protein)	Glutathione Reductase Activity (Unit/mg protein)
***C. edulis***	0.80 ± 0.02^a^	26.66 ± 0.22^a^	1.81 ± 0.87^a^	0.17 ± 0.03^a^
***P. capensis***	0.41 ± 0.10^b^	49.36 ± 0.03^b^	3.28 ± 0.13^b^	0.45 ± 0.04^b^

Results are expressed as mean ± SEM. Means with the same superscript along the column are not significant at p > 0.05 by one way ANOVA followed by Tukey's post hoc test.

On other hand, a significant difference was observed in superoxide dismutase activities between *C. edulis* and *P. capensis* extracts (p < 0.05; Table 7). As shown in Table 7, the leaf extract of *C. edulis* had SOD activity of 7.89 ± 0.87 unit/mg protein whereas that stem bark extract of *P. capensis* had SOD activity of 12.72 unit/mg protein. As depicted in Table 7, the leaf extract of *C. edulis* had a glutathione reductase activity of 0.17 unit/mg protein, whereas the stem bark extract of *P. capensis* exhibited a glutathione activity of 0.45 mg/ml.

### 4.5 Identification of phytocompounds in aqueous, EtOAC and DCM extracts of *C. edulis* and *P. capensis*

**Fig. 9:**
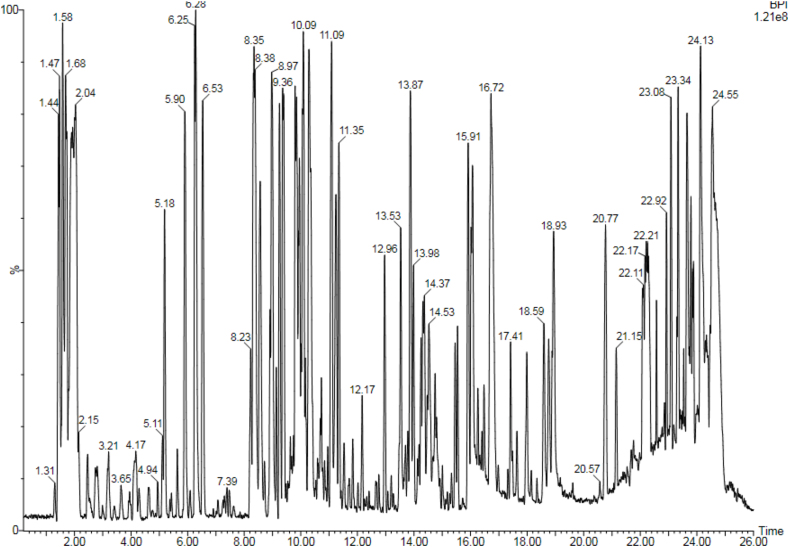
LC-MS chromatogram of aqueous leaf extract of *C. edulis*. Retention times of compounds are shown above peaks.

**Fig. 10:**
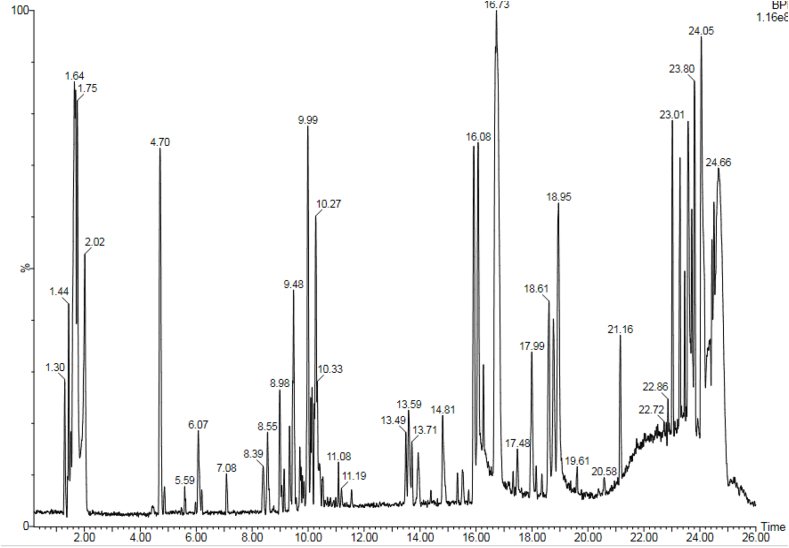
LC-MS chromatogram of aqueous stem bark extract of *P. capensis*. Retention times of compounds are shown above peaks.

**Fig. 11:**
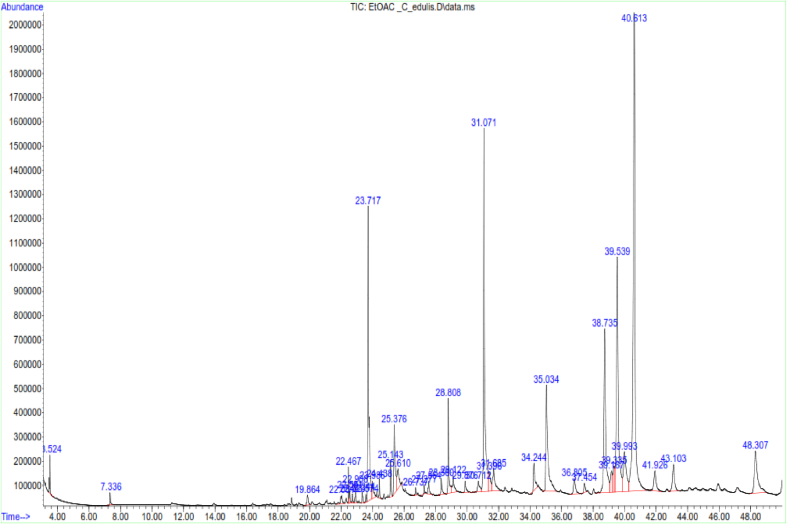
GC-MS chromatogram of ethylacetate leaf extract of *C. edulis*. Retention times of compounds are shown above peaks.

**Fig. 12:**
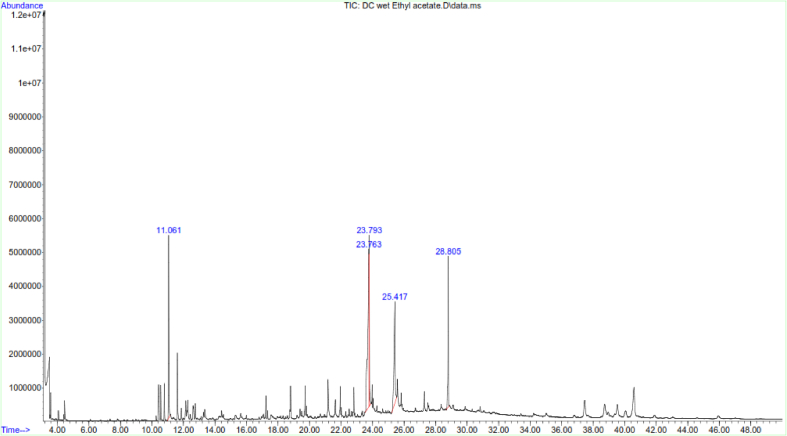
GC-MS chromatogram of ethylacetate stem bark extract of *P. capensis*. Retention times of compounds are shown above peaks.

**Fig. 13:**
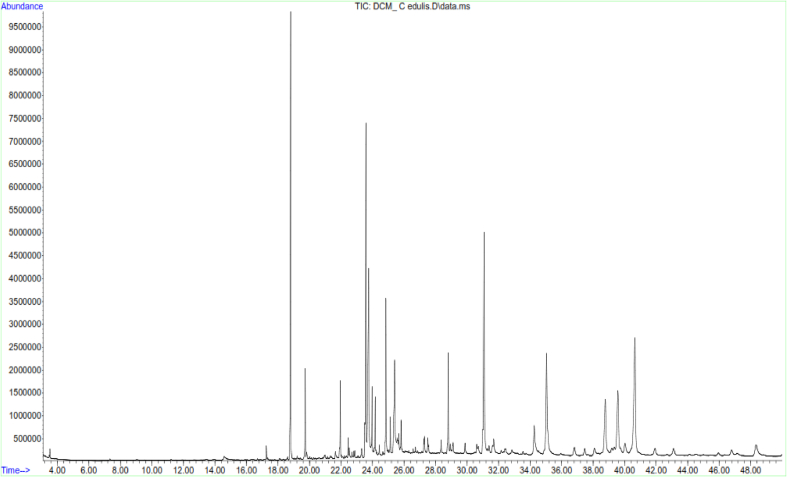
GC-MS chromatogram of dichloromethane leaf extract of *C. edulis*. Retention times of compounds are shown above peaks.

**Fig. 14:**
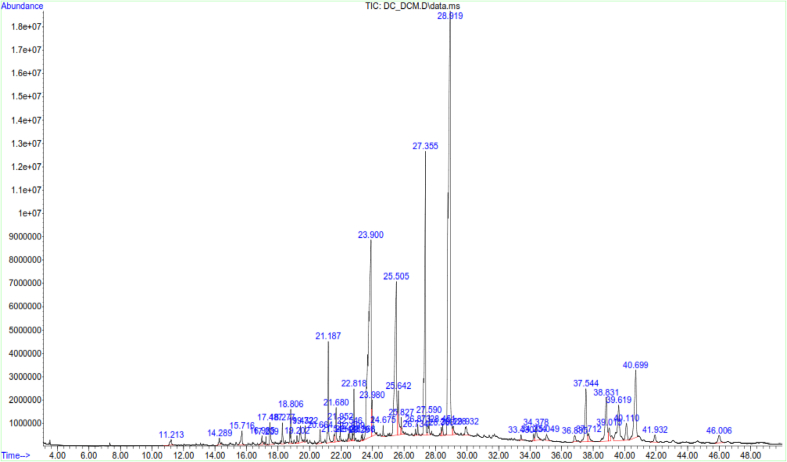
GC-MS chromatogram dichloromethane stem bark extract of *P. capensis.* Retention times of compounds are shown above peaks.

LC-MS and GC-MS analyses of aqueous, EtOAc and DCM extracts of *C. edulis* and *P. capensis* revealed presence of phytoactive compounds that have been associated with antioxidant and radical scavenging activities (Tables 8–13). LC-MS spectra for aqueous extracts of *C. edulis* and *P. capensis* are shown in Figs. 9 and 10 whereas GC MS spectra of ethylacetate and dichloromethane extracts are illustrated in Figs. 11–14.

**Table 8:** LMCS analysis of quantitative phytochemical composition of aqueous leaf extract of *C. edulis*

Retention Time (min)	Compound Name	Conc. (μg/mg)	% Abundance
1.31	Catechin	0.007	0.34
1.44	Epichatechin	0.066	3.18
1.54	4-hydroxycinnamonic acid	0.006	0.29
1.58	Citric acid	0.120	5.78
2.04	P-coumaric acid	0.012	0.58
4.94	Rutin	0.006	0.29
6.28	Dicaffeoylquinic acid	0.113	5.44
6.53	Quercetin-3-O-glycosyl-xyloside	0.100	4.8
9.13	Quercetin-3-O-Rhamnoside	0.024	1.16
9.39	Apigenin	0.071	3.42
9.42	Luteolin	0.091	4.38
9.48	Quercetin	0.002	0.1

**Table 9:** LCMS analysis of quantitative phytochemical composition of aqueous stem bark extract of *P. capensis*

Retention Time (min)	Compound Name	Conc. μg/mg)	% Abundance
1.30	Catechin	0.021	5.95
1.40	Epichatechin	0.020	5.67
1.51	4-hydroxycinnamonic acid	0.014	4.00
1.69	Quercetin	0.038	10.76
4.70	Rutin	0.093	26.03
8.61	Quercetin-3-O-rhamnoside	0.038	10.76
			
9.14	Kaempferol	0.005	1.42
9.33	Lutelion	0.014	4.00
9.45	Apigenin	0.011	3.12
9.57	Ellagic acid	0.001	0.28
20.18	P-coumaric acid	0.098	27.76

**Table 10:** GCMS analysis of quantitative Phytochemical Profiles of the EtOAc Extracts of *C. edulis*

Retention Time (min)	Compound Name	Conc. (μg/mg)	% Abundance
0.44	Stigmasterol	0.44	1.56
7.36	Limonene	0.1	0.35
16.85	Menthol	0.2	0.71
21.82	Tetradecanoic acid	0.07	0.25
23.71	Hexadecanoic acid orpalmitic acid	2.66	9.43
25.14	Phytol	0.46	1.63
25.38	Oleic acid	1.15	4.08
25.61	Octadecanoic acid or stearic acid	0.92	3.26
31.07	Squalene	4.0	14.10
33.56	β-Tocopherol	0.15	0.53
35.56	α-Tocopherol	2.97	10.53
36.81	Campesterol	0.68	2.41
38.74	β –Sitosterol	3.23	11.46
39.33	α-Amyrin	1.5	5.43
40.61	β-Amyrin	9.14	32.10

**Table 11:** GCMS analysis of quantitative phytochemical composition of the EtOAc Extract of *P. capensis*

Retention Time (min)	Compound Name	Conc. (μg/mg)	% Abundance
12.87	p-Cymene	0.2	0.38
13.75	Camphor	0.1	0.19
17.17	Copaene	0.44	0.84
22.45	Phytol	0.6	1.15
23.79	Hexadecanoic acid or palmitic acid	21.54	41.4
24.26	Oleic acid	0.70	1.35
25.42	6-Octadecanoic acid	11.66	22.40
25.58	Octadecanoic acid or stearic acid	3.70	7.11
33.54	β-Tocopherol	0.27	0.52
34.89	γ –Tocopherol	0.25	0.48
35.02	α-Tocopherol	0.80	1.53
36.80	Campesterol	0.40	0.77
37.46	Stigmasterol	2.00	0.38
38.73	Stigmast-7-en-3-ol	1.96	3.77
39.53	β-Amyrin	1.7	3,27
39.37	α-Amyrin	0.38	0.73
41.90	Stigmast-4-en-3-one	0.42	0.81
45.94	Betulin	0.61	1.17

**Table 12:** GCMS analysis of quantitative Phytochemical Composition of the DCM Extract of *C. edulis*

Retention Time (min)	Compound Name	Conc. μg/mg	% Abundance
17.17	Copaene	0.1	0.12
17.78	Caryophyllene	0.1	0.12
23.75	Hexadecanoic acid or palmitic acid	10.3	18.74
25.13	Phytol	2.1	3.82
25.40	Oleic acid	6.6	12.00
31.08	Squalene	9.9	18.01
33.55	β-Tocopherol	0.7	1.27
33.75	γ –Tocopherol	0.4	0.73
35.05	α-Tocopherol	9.6	17.46
36.81	Campesterol	1.5	2.73
37.46	Stigmasterol	1.0	1.81
38.77	Stigmast-8(14)-en-3.beta.-ol	6.5	11.82
39.35	α-Amyrin	0.9	1.75
39.57	β-Amyrin	7.00	12.73
40.66	Taraxasterol	12.7	23.01
45.97	Betulin	0.6	1.09

**Table 13:** GCMS analysis of quantitative Phytochemical Composition of the DCM Extract of *P. capensis*

Retention Time (min)	Compound Name	Conc. μg/mg	% Abundance
13.77	Bornanone or camphor	0.10	0.10
17.16	A-copaene	0.25	0.25
22.45	Phytol	1.85	1.85
23.91	Hexadecanoic acid	42.9	42.48
25.01	6-0ctadecenoic acid	31.97	31.65
25.64	Octadecanoic acid or stearic acid	6.16	6.10
31.07	Squalene	2.09	2.07
33.55	ὃ-Tocopherol	1.65	1.63
35.05	α-Tocopherol	1.22	1.20
36.84	Campesterol	0.39	0.39
37.54	Stigmasterol	1.31	1.30
38.58	β-Sitosterol	0.21	0.21
39.62	β-Amyrin	4.65	4.60
40.70	Α-Amyrin	5.09	0.50
41.93	Stigmast-4-en-3-one	1.71	1.69
46.01	Betulin	0.42	0.42

## 5 Discussion

The human body ensures a cellular redox state by maintaining a balance between free radical formation and elimination. However, an imbalance that favors pro-oxidant production results in oxidative stress, which is pivotal in progression of chronic ailments. Antioxidants have the ability to react with and eliminate oxidizing free radicals, which inhibit cellular damage [24]. Synthetic antioxidants can improve the capacity to ameliorate oxidative stress in the human body. However, their applications are limited by adverse effects and relatively prohibitive costs. Therefore, studies are being undertaken aimed at assessing the efficacies and safety of naturally occurring plant-based antioxidant compounds [25]In this study, it was established that *C. edulis* and *P. capensis* extracts have remarkable *in vitro* antioxidant activities.

*In vitro* assays of the extracts were assessed through ferric reducing activity (FRAP), iron chelating capacity, hydroxyl and DPPH radical scavenging activities. Moreover, the enzymatic activities of catalase, glutathione reductases and SOD were assessed. It was established that DPPH and hydroxyl radical scavenging activities, FRAP and chelation effects of *C. edulis* and *P. capensis* extracts were concentration dependent. This could be attributed to increasing concentrations of phytocompounds with antioxidants activities in the extracts. The findings are in tandem with those demonstrated by Adebiyi et al. [26], whereby ethanol stem and leaf extracts of *Grewia carpinifolia* showed concentration-dependent ferric reducing activities of*.* Iron chelating activities of organic extracts of *Pandanus pygmaeus* were also shown to occur in a similar manner [27]. Concentration-depedent DPPH radical scavenging and FRAP activities of *Vernonia lasiopus* have also been reported by Guchu et al. [28].

With regards to free radical scavenging activities, IC_50_ values for majority of *C. edulis* and *P. capensis* extracts were found to be between 101 and 150 μg/ml, thereby implying radical scavenging activities [29]. Nevertheless, there were variations in antioxidant activities of the plants extracts, whereby the leaf extracts of *C. edulis* had more pronounced antioxidant activities, compared to stem bark extracts of *P. capensis.* Perhaps this could be because of the complex compositions and synergistic interactions of individual phytocompounds present in each extract [30].

Antioxidants exert their function through diverse mechanisms viz. reducing power, chelating transition metal, radical scavenging activities, and disintegrating radicals [31]. Reducing effects of *C. edulis* and *P. capensis* extracts were evaluated using FRAP assay. This was done by directly determining the abilities of the extracts to reduce ferric to ferrous ion (FRAP), accompanied by the formation of an intense blue-coloured Fe^2+^ (Perl's Prussian blue ferric ferrocyanide) [32]. Reduction of ferric ions occurs through donation of electron or hydrogen atoms [33]. Therefore, the reducing property of *C. edulis* and *P. capensis* extracts suggests presence of reductants, which facilitated interruptions of the radical chain. The FRAP assay is significant because in biological systems, antioxidants with reducing powers are able to neutralize lipid peroxyl radicals, thereby converting the radicals into stable molecules [24,34].

Extracts of *C. edulis* and *P. capensis* extracts managed to chelate ferrous ions, as shown by the reduction in the red-coloured Fe^2+^-ferrozin complex, suggesting that the compounds captured the ferrous ions before ferrozine. Excess Fe^2+^ ions accelerate ROS formation, leading to cellular injury. Mechanistically, through Fenton chemistry, Fe^2+^ reacts with H_2_O_2_ to generate hydroxyl radicals [34]. In addition, Fe^2+^ accelerates formation of alkoyl and peroxy radicals. Therefore, by binding metal ions, effective iron chelators stabilize transition metals, thereby inhibiting lipid peroxidation and the subsequent iron-mediated oxidative stress [35].

Radical quenching abilities of *C. edulis* and *P. capensis* were assessed by evaluating their effects on hydroxyl and DPPH radicals. The scavenging capacity of extracts towards hydroxyl radicals was based on degradation of d-ribose by hydroxyl radicals in a Fenton reaction. Scavenging activities were indicated by a decrease of the pink chromogen [4]. Hydroxyl radicals, which are the most reactive reduced forms of dioxygen, are directly implicated in irreversible DNA damage. They oxidize guanosine to 8-hydroxyl-2-deoxyguanosine, which modifies DNA, leading to carcinogenesis [36]. Moreover, they cause damage to lipids and proteins, resulting in oxidative stress-related diseases, including cancer [4].

As a proof that the plant extracts can scavenge different free radicals in various systems, *C. edulis* and *P. capensis* extracts caused neutralization of DPPH radicals that are stoichiometrically associated with the presence of proton radical scavengers. DPPH, a stable radical, accepts hydrogen atoms or electrons to form a diamagnetic molecule [17]. The reaction is accompanied by a colour change, from deep violet to yellow. Therefore, it can be postulated that the *C. edulis* and *P. capensis* extracts exerted their DPPH scavenging effects by reducing the DPPH radical to the corresponding hydrazine compound, perhaps by their hydrogen donating abilities. In biological systems, compounds with DPPH radical scavenging activities donate hydrogen atoms to the radicals, more so the hydroperoxide and lipid peroxides radicals that are propagators of radical chain autoxidations of lipids [31].

Through further assessments, *C. edulis* and *P. capensis* extracts showed potent catalase, SOD and glutathione reductase activities, proving their ability to disintegrate and eliminate free radicals. Such antioxidant enzymes serve as intrinsic defense tools against oxidative damage. Catalase, a porphyrin containing enzyme, dismutases H_2_O_2_ to water and oxygen molecules [37]. H_2_O_2_ is a protonated form of superoxide radical that is generated by dismutation of superoxide anions by SOD [38]. Hydrogen peroxides cause DNA modification by inducing strand breaks and DNA cross links. In addition, they cause the modification of pyrimidines, purines and deoxyribose [39]. In Fenton reactions, Fe^2+^, ion of transition metal can donate an electron to H_2_O_2_ resulting in its decomposition, consequently producing hydroxyl radicals. The resultant hydroxyl radical has the ability to react with DNA, lipids and other molecular targets, promoting cellular damage [38].

Upon exposure to *P. capensis* and *C. edulis* extracts, the superoxide dismutase (SOD) activities were demonstrated by a reduction in blue coloured formazan, which was due to inhibition of nitroblue tetrazolium (NBT). The enzyme catalyzes the breakdown of superoxide radicals to H_2_O_2_ and oxygen. During aerobic metabolism, superoxide radicals are constantly produced and are incapable of directly inducing DNA damage. However, superoxide anions can generate hydroxyl radicals that mediate DNA damage [40]. In their prooxidative states, ions of transition metals can catalyze the formation of DNA oxidants, resulting in further DNA damage. In addition, superoxide acts on lipid membranes, initiating the release of lipid radicals that result in membrane lipid peroxidation [40,41]. Therefore, SOD plays a role in cellular antioxidant defense systems.

Glutathione activities of *C. edulis* and *P. capensis* extracts were indicated by a decrease in absorbance, a reflection of reduction of GSSG during coupled with NADPH oxidation by GR in the extracts [10]. Glutathione reductase (GR), a ubiquitous enzyme, maintains a pool of reduced cellular glutathione, a non-enzymatic antioxidant, through the glutathione redox cycle. In addition, GSH maintainsis involved redox homeostasis thereby, protecting cells from toxicity of xenobiotic electrophiles and oxidative damage by reacting with organic peroxides and free radicals.

Preceding research has reported antioxidant enzymatic activities of plants have. For instance, a Kumar et al. [10] reported significant SOD activities in *Acacia catechu*, *Cinnamomum cassia* and *Citrus limon* extracts, whereas Shams et al. [11] demonstrated that *Calamintha officinalis*, an aromatic herb, had SOD activities. Kumar et al. [10] reported catalase activities of *C. fistula*, *A. catechu*, *C. cassia* and *C. limon* extracts. A study on antioxidant and free radical scavenging capacities reported specific catalase activities of *Caralluma flava* extract [42]. Furthermore, Rajan and Pushpa [12] reported high glutathione reductase activities in methanol leaf and seed extracts of *Momordica charantia* and *Syzygium cumini*. Additionally, assessment of GR activities revealed that they were significantly high in *C. fistula* and *C. limon* extracts [10].

The antioxidant activities of *C. edulis* and *P. capensis* extracts can be ascribed to a repertoire of secondary metabolites present in the extracts. Flavonoids including apigenin, quercetin, rutin, lutelion, catechin and kaempferol were found present in *C. edulis* and *P. capensis* extracts. Flavonoids have been reported to have antioxidant properties. *In vitro* iron (II) chelating activities of bark and fruit extracts of *Tetrapleura tetraptera* were largely associated with the presence of quercetin and apigenin, which have dihydroxyl groups that donate electrons to ferrous ions thereby, stabilizing Fe^2+^ [43]. Moreover, the DPPH radical scavenging activities of quercetin, catechin, kaempferol, lutelion, catechin and rutin in *Lepidium sativum* and *Raphanus sativus* have been reported. These studies concluded that the effects of flavonoids on DPPH radicals were due to their proton donating abilities [44,45]. Lalhminghlui et al. [46] elucidated the hydroxyl radical scavenging activities of flavonoids, including catechins which are attributed to their proton donating abilities.

Terpenes, including squalene, phytol, α and β amyrin may also be associated with the observed antioxidant activities of *C. edulis* and *P. capensis* extracts. Amarowicz [47] reported that squalene had DPPH radical scavenging activities, which was attributed to its ability to donate hydrogens or electrons. In addition, its hydrogen donating capacity has been demonstrated in generation of GSH from its oxidized form, GSSG.

With regards to phytol, Santos et al. [48] reported scavenging activities of phytol for hydroxyl radicals as well as nitric oxide. Phytol has a high scavenging capacity for ABTS and DPPH radicals, which are attributed to its hydrogen-atom donating ability. Similarly, α and β amyrin are associated with antioxidant activities. For example, α and β amyrin isolated from dichloromethane extracts of *Myricianthes pungens* donated protons to DPPH radicals, thereby neutralizing them [49].

Limonene may also have been involved in the antioxidant activities of *C. edulis* extracts [50]. Limonene exerts its free radical scavenging activities by donating hydrogen or electrons, thereby breaking radical chain reactions [50,51]. Moreover, limonene enhances SOD and catalase expression [52]. In rat models, limonene upregulated glutathione S-transferase and induced the activities of glutathione peroxidase in the oesophagus [51]. This may have been attributed to limonene-mediated increase in expressions of genes encoding SOD and CAT enzymes [53] Research on antioxidant activities of *Mentha piperita* extracts revealed the main compound was menthol, which conferred hydrogen donating abilities to the extract [54].

This study postulates that tocopherols contributed to the antioxidant activities of *C. edulis* and *P. capensis* extracts. Vitamin E exerts inhibits lipid peroxidation chains by intercepting peroxyl radicals (LOO^.^) by donating a hydrogen atom [55]. Upon hydrogen atom donation, the compound becomes unreactive since the unpaired electron on the oxygen is primarily delocalized into the aromatic structure, increasing its stability. Still, it can further be oxidized into α-tocopheryl quinine [56].

Phytosterols, including β-sitosterol, may also have been involved in the antioxidant activities of *C. edulis* and *P. capensis* extracts. As a modest ROS scavenger, β-sitosterol was shown to reverse glutathione/reduced glutathione ratio impairment in Phorbolmyristate acetate stimulated RAW 264.7 macrophages cells [57]. This effect was correlated with increased glutathione peroxidase and manganese SOD activities. In addition, Manish et al. [58] validated the antioxidant potential of β-sitosterol based on their *in vitro* experiemental study of *Eulophiaochreta* and *Eulophiaherbacea.* This compound has reducing properties and DPPH free radical scavenging activities whereby it readily donates protons to radicals. Stigmasterol from *B. monosperma* enhanced the activities of SOD, catalase and glutathione and suppressed hepatic lipid peroxidation, suggesting that it exerts its effect by upregulating the expression levels of enzymatic antioxidants [53,59].

## 6 Conclusion

In this study it was established that the extracts of *C. edulis* and *P. capensis* antioxidant activities. The activities are attributable to presence of phytocompounds, which have the ability to donate hydrogen atoms or electrons, thereby quenching the radicals. Furthermore, the phytocompounds are associated with increased expression levels of enzymatic antioxidants. From the findings, antioxidant properties of *C. edulis* and *P. capensis* extracts could be one of the underlying mechanisms of their therapeutic activities. Nevertheless, there is a need for further purification and characterization of phytochemicals associated with anticancer and antioxidant activities.

### Author contribution statement

Carolyn Wanjira Muruthi: Conceived and designed the experiments; Performed the experiments; Analyzed and interpreted the data; Wrote the paper.

Mathew Piero Ngugi: Analyzed and interpreted the data.

Steven Runo, Peter Mwitari: Contributed reagents, materials and analysis tools.

### Funding statement

This research did not receive any specific grant from funding agencies in the public, commercial, or not-for-profit sectors.

### Data availability statement

Data will be made available on request.

### Declaration of interest's statement

The authors declare no competing interests.
